# Diferencias en la estimación del filtrado glomerular usando la nueva fórmula eGFRcr CKD EPI AS 2021 vs. eGFRcr CKD EPI 2009

**DOI:** 10.1515/almed-2022-0024

**Published:** 2022-07-18

**Authors:** Cristian Ríos Campillo, María P. Sanz de Pedro, Sara Aldana Barcelo, María Auxiliadora Bajo Rubio, Antonio Buño Soto, Rubén Gómez Rioja

**Affiliations:** Servicio de Análisis Clínicos, Hospital Universitario La Paz, Madrid, España; Servicio de Nefrología, Hospital Universitario La Paz, Madrid, España

**Keywords:** ecuación CKD EPI 2009, eGFRcr CKD EPI AS 202, filtrado glomerular estimado (eGFR)

Estimado Editor,

El cálculo del filtrado glomerular estimado (eGFR) a partir de la creatinina sérica forma parte de la práctica clínica habitual para evaluar la función renal y el ajuste de dosis de ciertos fármacos. Las últimas guías de práctica clínica recomiendan el uso de la ecuación CKD EPI 2009 [1, 2], cuyo uso es apoyado también de forma conjunta por la Sociedad Española de Nefrología y la Sociedad Española de Medicina de Laboratorio [3].

Una de las variables usadas como factor corrector en el eGFR con esta fórmula es la raza, asumiendo que, en relación a la masa muscular en la “raza negra”, los valores de creatinina son mayores que en la “raza no-negra”. El término raza presenta una connotación más social que biológica, por lo que su inclusión en el cálculo del eGFR ha sido objeto de debate [4], [5], [6].

La cistatina C es un marcador de filtrado glomerular alternativo que no se ve influenciado por la masa muscular. Sin embargo, en relación a la creatinina, los métodos de medida están peor estandarizados y además no está exenta de factores extra renales que condicionan su concentración sanguínea. Pese a esto, su uso está recomendado en el cálculo del eGFR, aislada (eGFRcys CDK EPI 2012) o combinada con creatinina (eGFRcr cys CKD EPI 2012), cuando la creatinina por sí sola presente limitaciones [7].

Recientemente, el grupo CKD EPI ha presentado nuevas ecuaciones del eGFR a partir de creatinina y cistatina C [8], modificando las constantes y eliminando el factor de la raza. El grupo recomienda la utilización de la nueva ecuación modificada y promociona el uso de la cistatina C como marcador no afectado por la masa muscular que, además, ofrecería un mejor ajuste usando la ecuación combinada.

Debido a que estas nuevas fórmulas están valoradas en una población con un alto porcentaje de pacientes de “raza negra”, creímos conveniente valorar la potencial repercusión en nuestra población. Para ello, se recogieron 14.539 resultados de creatinina enzimática (método creatininasa en Atellica Solution^®^ CH Analyzer estandarizado conforme al SRM967) de pacientes procedentes del servicio de nefrología, y se recalculó el eGFR usando la nueva ecuación eGFRcr CKD EPI AS 2021. En nuestro entorno, el factor corrector de la raza no se ha tenido en consideración a la hora de aplicar la fórmula en uso (eGFRcr CKD EPI 2009) dada la subjetividad del término a la hora de recogerlo en la historia clínica, pudiendo evaluar la función renal del paciente con métodos alternativos como el aclaramiento de creatinina en orina de 24 h o haciendo uso de la cistatina C.

La comparación de eGFR presentó un sesgo constante y proporcional de +1.82% con la nueva ecuación eGFRcr CKD EPI AS 2021 en relación a la anterior, siendo necesario reclasificar en promedio el 10, 3% de los resultados entre los diferentes estadios de enfermedad renal crónica de la guía KDIGO 2012 (Tabla 1). De forma llamativa, un 17, 4% de los resultados de pacientes en estadio G3a (eGFR 45–59 mL/min) se reclasificaron en estadio G2 (eGFR 60–89 mL/min) y un 12, 4% de los pacientes en estadio G4 (eGFR 15–29 mL/min) se reclasificaron en estadio G3b (eGFR 30–44 mL/min), puntos de corte habituales en la derivación del paciente al nefrólogo y en el ajuste de dosis de fármacos respectivamente.

**Tabla 1:** Reclasificación de resultados en estadios KDIGO (G1-G5) comparando ecuación eGFRcr CDK EPI 2009 y eGFRcr CKD EPI 2012.

Cambio de estadio	Nº resultados de pacientes en cada estadio según eGFRcr CKD EPI 2009	Nº resultados reclasificados	% Resultados reclasificados
G2 → G1	1.865	187	10.0%
G3a → G2	1.934	335	17.4%
G3b → G3a	2.673	377	14.1%
G4 → G3b	3.117	386	12.4%
G5 → G4	3.819	211	5.5%
Total	14.539	1.497	10.3%

Otros estudios han comparado la capacidad de clasificación de esta nueva ecuación en relación al filtrado glomerular medido a través del aclaramiento de iotalamato, obteniendo resultados similares a los obtenidos en nuestra aproximación [9].

Los datos presentados exhiben varias limitaciones, principalmente el sesgo de selección al usar exclusivamente pacientes en seguimiento por Nefrología, no haber tenido en cuenta la albuminuria para el estadiaje ni haber considerado la raza como variable en la ecuación en uso. La intención de esta aproximación es prever lo que sucedería en nuestra población si implantásemos la nueva ecuación de eGFRcr CKD EPI AS 2021.

En cuanto a la implementación de estas nuevas ecuaciones, sería necesaria la evaluación de las nuevas fórmulas frente al filtrado glomerular medido en nuevas cohortes, deseablemente en una población similar a la nuestra. Dada la dificultad de llevar a cabo este procedimiento en la práctica clínica, sería recomendable que cada laboratorio evaluara en su población el impacto del cambio. También hay que contemplar la existencia de otras ecuaciones recomendadas en guías clínicas actuales [2, 10], el uso de otros marcadores como la cistatina C, o la recogida de orina de 24 h como alternativas para completar la evaluación de la función renal.
